# Gamification and education: A pragmatic approach with two examples of implementation

**DOI:** 10.1017/cts.2021.806

**Published:** 2021-06-28

**Authors:** James H. Willig, Jennifer Croker, Lisa McCormick, Meena Nabavi, Jeremey Walker, Nancy P. Wingo, Cathy C. Roche, Carolyn Jones, Katherine E. Hartmann, David Redden

**Affiliations:** 1 School of Medicine, University of Alabama at Birmingham, Birmingham, AL, USA; 2 Center for Clinical and Translational Science, University of Alabama at Birmingham, Birmingham, AL, USA; 3 School of Public Health, University of Alabama at Birmingham, Birmingham, AL, USA; 4 School of Nursing, University of Alabama at Birmingham, Birmingham, AL, USA; 5 College of Nursing, The Ohio State University, Columbus, OH, USA; 6 Vanderbilt Institute for Clinical Translational Research, Vanderbilt University Medical Center, Nashville, TN, USA

**Keywords:** Gamification, education, games, pedagogy, adult learners, compliance training

## Abstract

Leveraging elements of game design and theories of human motivation, gamification provides a variety of techniques to engage learners in novel ways. Our Clinical and Translational Science Award created the software platform (Kaizen-Education©) to deliver gamified educational content in 2012. Here, we explore two novel use cases of this platform to provide practical insights for leveraging these methods in educational settings: (1) national training in rigor, reproducibility, and transparency and (2) attainment of learner competency (n = 7) as a gauge of curricular effectiveness across Master of Public Health degree tracks (n = 5). Data were captured in real time during player interaction with Kaizen-Education© to provide descriptive analyses of player engagement in both implementation examples. We then assessed item analysis to assess knowledge gain and competency attainment. We have just begun to leverage the potential for gamification to engage learners, enhance knowledge acquisition, and document completion of training, across various learning environments. We encourage a systematic approach to gamification applying insights from self-determination theory to learners and learning environments, a methodical approach to game design and rigorous analysis after implementation to generate evidence-based insights to maximize educational return for time invested.

## Introduction

Games are competitive, focused on winning, and characterized by structured, rule-based play. Serious games carry a dual purpose, to teach and remain entertaining or engaging while collectively addressing a scientific or societal challenge. What then is gamification? Gamification is a design technique. Gamification is the application of game design elements in any nongame context, such as a learning activity or course, facilitating achievement of learning objectives and enhancing learner engagement [1]. For example, game design elements such as scoreboards or badges (physical or online) can serve to provide visual markers of learner progress or to reward achievement, while increasing engagement and motivation by allowing learners to interact with the learning experience.

Self-determination theory provides a lens for analyzing gamification. This theory postulates that learner motivation is in one of three states (amotivation, intrinsic, or extrinsic motivation), each with a “regulation” essential to how motivation is achieved, and that intrinsic motivation outperforms extrinsic motivation leading to more effective learning [2]. Importantly, motivation for an activity can move from extrinsic to intrinsic by fulfilling the psychological needs for competency, autonomy, and relatedness [3–5]. Gamification, through tools like goal setting, learner control, and engagement, can address these psychological needs and become a fulcrum for spurring a learner to the intrinsic part of the motivational spectrum [4, 6, 7].

Leveraging elements of game design and theories of human motivation, gamification provides a wide range of techniques to engage learners in novel ways. Engagement is not confined to classic educational environments (classrooms, medical rounds), as digital gamification strategies untether teaching from tradition, providing new educational opportunities. Here, we present two unique experiences implementing gamification in non-traditional ways. First, we describe the use of gamification to teach rigor, reproducibility, and transparency to investigators across multiple institutions nationwide. Next, we detail the use of gamification to gauge learner attainment of public health competencies at the conclusion of their Master’s in Public Health training and explore the use of that data to monitor curricular effectiveness. Our goal is to provide practical insight into applying gamification principles.

## Methods

### Setting, Participants, and Software Platform

In 2012, at the University of Alabama at Birmingham (UAB), we began the development of a software platform to deliver gamified educational content to learners. Development and implementation of the software were supported by our Center for Clinical and Translational Science (CCTS, UAB’s clinical translational science award, UL1TR003096). We named our platform Kaizen-Education, drawing from the Japanese word meaning “continuous improvement” as it resonates with the principle of “lifelong learning” we seek to imbue in learners. Our vision was to create a software platform that enabled educators to leverage gamification to engage learners through question-driven and knowledge-based competitions. The Kaizen-Education platform has been used across multiple settings including graduate and undergraduate/graduate medical and nursing education, education in research methods and even patient education [8–14]. While reports exist on the use of gamification to enhance focused educational settings, we report on two novel uses of our gamification platform to display areas of untapped potential of these techniques. First, we report on the use of gamification to expand a local training opportunity in rigor, reproducibility, and transparency (R2T) for those pursuing biomedical research to a national audience. Second, we describe the use gamification to assess learner competency attainment across the five Master of Public Health (MPH) educational tracks as a gauge of curricular effectiveness. Finally, drawing on these two examples and our prior experience, we share insights into our approach to gamification providing pragmatic insights into its utilization. The UAB institutional review board protocol approved our research (Protocol Number IRB-121204006).

### Leveraging Gamification within Kaizen-Education and Beyond

We encourage instructors considering implementing gamification as a learning activity to consider the Learning Mechanics-Game Mechanics (LM-GM) Model, proposed by the Games and Learning Alliance (GALA, www.galanoe.eu). While created to foster the design of pedagogy-driven serious games, we find elements of the proposed framework helpful in planning the implementation of gamification. The LM-GM model acknowledges that learning is a complex activity investigated and modeled through several pedagogical theories and approaches including behaviorism, cognitivism, humanism, personalism, constructivism, etc. The LM-GM asks the game creator to define learning objectives, then to consider which learning mechanics (spaced repetition, pre-testing, multi-directional emphasis, etc.) may be employed to enhance teaching of a learning objective. Then, the instructor is asked to pair one or more game mechanics available within the gamification approach being used (badges, leaderboards, reminders/notifications, etc.) that will bolster each learning mechanic [15]. Thus, when using our software, we encourage game managers to first clearly delineate their learning objectives, then couple them with the learning mechanic and facilitating game mechanic within Kaizen-Education that will be leveraged to achieve it. Completing a table linking learning objectives with learning mechanics and game mechanics for whatever gamification approach or platform will be used using the LM-GM framework will provide educators strategic insight on how to optimize gamification to achieve specific learning objectives.

To gain access to the Kaizen-Education platform, an interested educator contacts UAB CTSA representatives (aguzman@uabmc.edu; ddempsey@uab.edu; jwillig@uabmc.edu) to be granted a game manager account. Game managers can access the Game Manager Portal, a website where game creators can formulate and build the structure of their gamified curriculum (adding players, questions, rewards, etc.). Training is available on demand, and users may also access shared resources (questions, badges, game structures) developed by other instructors within the Kaizen-Education community. Once a game is built in Kaizen-Education, players (learners) receive an invitation via their email accounts with a link to finalize their profiles. A learner’s profile allows them to access the game they have been invited to “play” either via the web or through the Kaizen-Education application (app), available for iOS and Android mobile operating systems.

A game manager typically starts game construction by setting general parameters (learning objectives, duration, use of teams, etc.). The next step is to create questions (single answer, multiple answer, containing pictures or videos, timed, or not timed, point value), then upload, and assign a release date for each one. Each question includes an explanation that can include text, photos, videos, or links to external resources/supplemental materials that relate to the learning objective. A game manager next decides which badge rewards to add to the game, the parameters for their attainment, and how many points to grant per badge collected. Common approaches, used as extrinsic motivators in some games, include individual leaderboards, level badges (awarded for accruing pre-determined point totals), and hotstreak badges (for achieving a pre-set number of consecutive correct responses). In addition, we have seen team leaderboards, marathon badges (reward for consistently completing questions on the day they are published for a pre-set number of days), and team badges (total team accuracy and total team participation) deployed as intrinsic motivators in other games. Further options exist within our software, but the key principle is that a game creator use their specific insight into their learners to balance extrinsic and intrinsic motivators to best engage them. To better understand the role of the game manager in planning, facilitating, and evaluating a game, we have created a checklist (see Table 1).

**Table 1.** Game Manager Checklist for Creating & Managing a Gamified Question Bank Curricula (within Kaizen-Education) and Applying the Learning-Mechanic/Game Mechanic Model

Plan & Create Game	Consider
Outline content to learning objectives (and competencies), consider learning mechanics and game mechanics to help achieve them	Map out what you plan to teach in advance. Carefully consider the learning mechanics you can use and the game mechanics you can leverage to enhance teaching
Develop questions and detailed answers (displayed for correct and incorrect responses) targeting learning objectives. Consider leveraging multi-directional emphasis during question creation to achieve learning objectives	Use different question types (choose all that apply, single best answer) and test various levels of Bloom’s Taxonomy in created questions (ex: remembering, understanding, applying, etc.)
Determine length of game (number of questions, delivered how often, sequence of questions)	Consider integrating learning mechanics like spaced repetition, interleaving, pre-testing, etc., as you sequence your questions to maximize learning
Determine the images and/or multimedia being used.	Has permission to use been acquired?
Determine if questions will be timed?	Applying to subset, all, or no questions
Determine number and source of players	Consider what you know about your learners and including them in game planning. Apply what you learn to select a combination of intrinsic and extrinsic rewards you will populate your game with to maximize retention and engagement. For example, we have found that Hot Streak & Level badges are reputational rewards that often appeal to extrinsically motivated learners, whereas team rewards & Marathon badges have appealed more to intrinsically motivated learners
Determine number of teams	
Generate naming conventions for game, teams, rewards	
Will you use reward badges and if so which types? o “Hot-streaks” (number of correct answers in a row) o “Levels” (number of correct answers) o “Marathons” (sequential days answering questions) o “Team Accuracy” or “Team Participation” rewards	
Design reward badges and plan when they should be awarded	Badges related to the topic(s) being taught are well received. Explicitly state reason reward badges achieved, and sequence them so they can be received throughout the game
Will there be external rewards at individual or team level?	Will there be real-world prizes? What is your budget for these?
Define strategy for engaging learners as players	Optional vs. required participation
Register users and assign them to teams (if teams being used)	If using teams, can you synergize with existing rivalries? Regional? Sports? Training level? Other?
Technology testing	Has software/game been tested on technology platforms available to learners?
Determine Human Subjects requirements (e.g., IRB and Informed consent requirements likely needed if using outputs for publication)	If research planned, attain IRB approval. Will you need informed consent? FERPA waiver applicable?^1^
**Manage Game**	
Create game announcements	Advertise! Make your game “an event” prior to starting. Reference prior effectiveness if done before and establish competitive tone
Launch & monitor game	Check that all users can access game. Check leaderboards daily and monitor participation
Plan frequency and content of motivational feedback – reports on leaderboards, etc.	Fan the flames of competition via periodic reports (email or in app) to keep users engaged. These serve as reminders and motivational tools when you highlight leaderboard data (best players, best teams, etc.) and often drive spikes in daily average users
Manage Game closure announcement and end date for access	Creating expectancy and a “countdown” to when game will close may augment competition and drive participation
**Conclude & Evaluate Game**	
Review and record final game metrics	Congratulate players and dispense pre-planned rewards
Provide overall feedback to players	Share overall usage statistics, thank the players, and celebrate collective achievements such as how many total questions were answered by the group
Review game utilization data	Including descriptive data on learners and on their interaction/utilization and engagement with your “game”
Solicit player satisfaction insights via survey or use more formal qualitative methods	Validate survey if needed. Employ formal qualitative methods to better ascertain learner experience and identify themes
Item analysis for continuous quality improvement	These methods allow detection of underperforming questions. Once these are identified, they can be modified for future games resulting in improved teaching materials
Disseminate findings	Publish. Please. The promise of gamification is great, and we must collectively document best practices for higher education

1 The Family Educational Rights and Privacy Act (FERPA) is a Federal law that protects the privacy of learner education records.

### Data Collection and Analyses

We describe two experiences using gamification to enhance training. The first is gamification of training in the scientific principles of rigor, reproducibility, and transparency (R2T). The second is the use of gamification to monitor competency attainment by learners completing a MPH degree.

Data collection for both examples used Kaizen-Education software. When a learner logged in and completed questions, all associated data were recorded (e.g., time, date, questions answered, accuracy of response, etc.). Independent variables for both analyses included overall game variables (e.g., total learners, teams, questions posted/completed, etc.) as well as participant level variables (e.g., institution, number of questions answered, timing of questions answered, badges earned, etc.). All data from learner interactions with Kaizen-Education software are housed on secure servers at UAB.

#### Gamification #1: Multi-institutional rigor, reproducibility, and transparency (R2T) training

Scientific progress requires the application of rigorous methodology as well as transparent reporting of methods, procedures, and outcomes that allow for independent replication [16]. To engage investigators in learning R2T principles to promote greater rigor, reproducibility, and transparency in the scientific community, our Biostatistics Epidemiology and Research Design group leveraged gamification to create the R2T game [9–11, 17]. The R2T game consists of 20 questions and takes a multimedia approach utilizing videos, and web links to manuscripts to teach important methodological principles.

The first pilot R2T game ran in January 2017 at UAB. Soon, investigators across our CCTS Partner Network began to participate. Ultimately, we offered an R2T game in partnership with Edge for Scholars (https://edgeforscholars.org/), extending this training to early-career investigators nationwide via social media outreach and registration. The R2T game is asynchronous; this enhances accessibility across time zones and disparate schedules, allowing investigators to build their R2T knowledge while competing with peers at other institutions. R2T games were administered throughout the year, and interested investigators can register anytime (https://www.uab.edu/ccts/training-academy/innovation/kaizen).

##### Analyses

We conducted descriptive analysis of all 21 R2T games to date detailing overall game characteristics (e.g., number of institutions represented, total number of players registered, number of questions posted, etc.) and player characteristics (e.g., completing players, play, accuracy, etc.). The initial incarnation of the R2T game ran from January 2017 to October 2019, and item analyses including total percent correct and point-biserial correlations were completed on those questions. The point-biserial correlation is a measure of how well a question can discriminate between test takers who know the subject matter and those that do not. Point-biserial correlations range from −1.0 to 1.0, positive values indicating that learners who performed well on that item did well on the global assessment indicating effective item discrimination. Item analysis results from January 2017 to October 2019 were used to reformulate some questions which were then deployed in subsequent games between January 2020 to November 2020 analysis for both periods is reported.

#### Gamification #2: Measuring attainment of public health competencies across degree tracks

To gauge learner mastery of School of Public Health competencies prior to graduation, a committee created a series of 79 multiple-choice questions in spring 2016. The committee included faculty who teach core courses in the MPH (Biostatistics, Management and Policy in Public Health Systems and Services, Fundamentals of Environmental Health, Social and Behavioral Sciences, and Introduction to Epidemiology). Questions probed concepts taught in each core course, and each was aligned to a specific core competency from seven institutionally determined school competencies (See Table 2). The game, including these multiple-choice questions, was played by learners over a 5-week period during MPH Capstone courses from the Fall 2016 to the Spring 2018 semesters. Learner performance in the game did not affect their MPH Capstone course grade. Play was incentivized by offering the team with the highest point total at the end of the game, a monetary prize for each team member. The three individual learners with the highest point totals received the school’s MPH Capstone Award, signifying their mastery of core competencies.

**Table 2.** Seven School of Public Health competencies tested during via gamification during the MPH capstone course Fall 2016 to the Spring 2018 semesters

Competency	Description	Questions
I	Apply design and analytical methods to describe, implement, evaluate, and interpret research addressing public health concerns	18
II	Identify how environmental and occupational hazards impact health	13
III	Apply legal and ethical principles in public health and research	8
IV	Communicate public health issues, research, practice, and intervention strategies effectively	11
V	Design public health programs, policies, and interventions, including planning, implementation, and evaluation	16
VI	Discuss the history and structure of public health systems	10
VII	Assess public health concerns in diverse cultures and communities	7

##### Estimation of competency

The mean score of all questions tied to a specific competency (See Table 2) was calculated globally and per MPH degree. The proportion of learners who achieved “competency” was also calculated. This was defined as correctly answering ≥ 50% and ≥ 70% of the questions tied to a specific competency and was calculated across the five MPH degree areas (Labeled A–E) for each of the seven core competencies.

## Results

### Multi-institutional Rigor, Reproducibility, and Transparency (R2T) Training

**Fig. 1.**
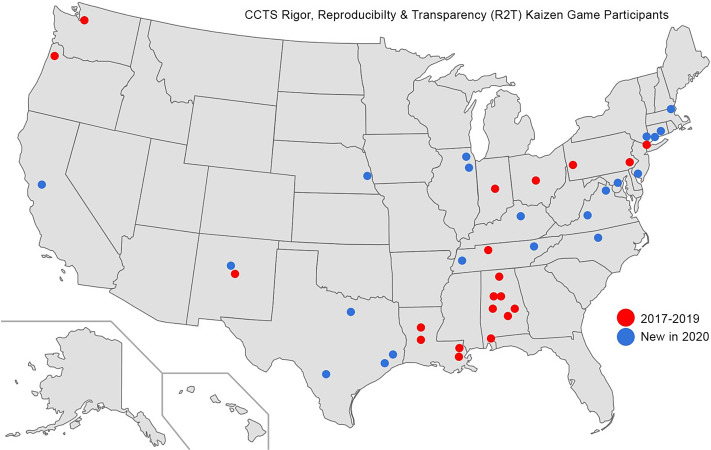
Geographic distribution of participants in Kaizen-R2T (Rigor, Reproducibility & Transparency) game 2017–2020.

In total, 27 Kaizen R2T games have been created. Six were either development games (played by game manager for software testing) or pilot games (played by recruited learners to test the system and provide feedback on question quality). We included 21 games deployed for early-career scholars (local and national, see Fig. 1) in the analyses. Overall, 595 learners from 41 institutions have registered for our R2T training. A total of 84% of players answered all 20 questions, and 92% of all posted questions were answered (See Table 3). For the 20 questions employed in the game during January 2017 to October 2019, the percent of individuals answering each specific question correctly ranged from 38% for a question concerning the definition of a p-value to 99% for a question focusing on sex as a biologic variable. For these 20 questions, the median value of the percent correctly answering a question was 87% with interquartile range of 18.5% (First Quartile = 75.4%, Third Quartile = 93.9%). For the 20 questions employed in the game during January 2020 to November 2020, the percent of individuals answering each specific question correctly ranged from 69% for a question concerning rigor of prior research to 99% for a question focusing on sex as a biologic variable. For these 20 questions, the median value of the percent correctly answering a question was 91% with interquartile range of 12.8% (First Quartile = 84.2%, Third Quartile = 97.0%). The range as well as the interquartile range of percent answering each question correctly was wider during January 2017 to October 2019 than for reformulated questions used from January 2020 to November 2020. Our point-biserial correlation range changed in the reformulated January 2020 – November 2020 questions (−0.10 to 0.37, median = 0.15) as compared to January 2017 to October 2019 (0.02 to 0.28, median = 0.14), indicating that the revised questions included new questions with better discrimination properties as well new questions with worse discrimination properties.

**Table 3:** Descriptive Characteristics of 21 Rigor, Reproducibility, and Transparency games administered on Kaizen-Education Software January 2017 through November 2020. Eleven (11) games were conducted from January 2017 to October 2019 using an initial set of questions. Another 10 games were conducted from February 2020 until November 2020 using a revised set of questions

Overall Game Characteristics	
Total Number of Unique Institutions having Learners Register	41
Total Number of Unique Learners Entering the Game	595
Total Number of Unique Learners Completing all 20 questions	501
Percent of Unique Players Completing all 20 questions	84.2%
Total Number of Questions Posted	11,900
Total Number of Questions Answered	10,894
Percent of Questions Answered	91.5%
Range of Question Difficulty (% of learners answering question correctly)	
January 2017 to October 2019	37.7% to 99.0%
January 2020 to November 2020	69.3% to 99.0%
Range of Point-Biserial Correlation for Questions	
January 2017 to October 2019	0.02 to 0.38
January 2020 to November 2020	−0.10 to 0.28

### Measuring Attainment of Public Health Competencies Across Degree Tracks

The competency assessment game was administered four times (fall 2016, spring 2017, fall 2017, and spring 2018) for 242 players. Students logged in frequently (2 ± 1 day intervals), and most questions were answered on day of release (74% ± 26%) or within 1–7 days from release (22% ± 20%). The largest cohorts were in tracks “B” and “D” of the MPH, with 56 and 80 participating learners, respectively. Learners logged in frequently (2 ± 1 day intervals), and most questions were answered on the day of release (74% ± 26%) or within 1–7 days from release (22% ± 20%). The mean global percentage of correct answers was 65% ± 11%.

We measured the proportion of learners reaching competency at two different thresholds: answering 50% or 70% of questions correctly for items related to a specific competency (See Table 2 for number of questions per competency) across five degree tracks (A–E). With a threshold of establishing competency by answering ≥ 50% of the associated questions correctly, the degree track with the highest percentage of learners achieving this metric was (Competency, Tracks, % accuracy): I (B, 79%), II (B and E, 86%), III (A, B, C and E, 100%), IV (B and E, 100%), V (B, 100%), VI (B and C, 98%), and VII (B, 93%). The proportion of learners achieving competency, defined as answering ≥ 70% of questions correctly, for each degree area was considerably lower: I (B, 39%), II (B, 27%), III (E, 82%), IV (B, 82%), V (B, 54%), VI (B, 91%), and VII (B, 57%).

## Discussion

Gamification improves learner performance, assessment outcomes, and longitudinal engagement [9–11, 17, 18]. Erosion of intrinsic motivation, high attrition rates, and a lack of effect on assessments have also been described among learners. A plethora of opportunities for exploring how and under what conditions gamification is most effective remain. We have presented two distinct uses of gamification. The first transformed a local initiative to improve research design and analysis by training investigators in rigor, reproducibility, and transparency, to a far-reaching effort available to investigators nationwide. The second was designed to gauge learner attainment of public health competencies at the conclusion of MPH training. These data led to the creation of a dashboard for comparisons of the effectiveness of competency training across our five MPH degree tracks. Those results provided insight into the effectiveness of curricula leading to discussions amongst faculty and leadership that spurred subsequent curricular adjustments. These experiences reveal the potential of gamification, both as a tool for enhancing individual learning, and for unlocking programmatic insights. We believe that leveraging the data produced when learners engage with digital gamification will uncover new strategies to optimize learner engagement, retention, and performance and will catalyze future widespread implementation of these techniques [1, 11]. We posit that a greater emphasis on educational analytics will guide the next generation of gamification applications.

Our R2T game has provided advanced research methodology training for 595 learners. These learners span the spectrum across multiple disciplines, and an unlimited geographic area. The availability of published questions completed asynchronously liberates learners to engage when they are available, not when a course schedule dictates. For each learning objective, expert faculty record explanatory videos, compose detailed answers, and/or add links to literature covering key teaching points that become available after a question is answered. Having this prepared content available offers us the opportunity to administer R2T games monthly, without impinging on limited teaching faculty time, while delivering high-quality educational materials. The elements of gamification, with learners competing in teams, earning badges, viewing their position relative to their peers in the leaderboards and other game design elements, contribute to engaging learners and propel many to complete the game.

An R2T qualification certificate is awarded to those clearing a pre-determined score threshold. This experience is notable in that gamification allowed a local initiative to grow to a regional initiative spanning our CTSA partner network and ultimately to a national one through partnership with Edge for Scholars at Vanderbilt. The scalability and potential for dissemination of digital gamification strategies are an exciting prospect for higher education. The lessons learned from analyzing the resulting data will provide educational analytics insights that will inform how best to engage learners and improve knowledge retention. Ultimately, such insights will guide educators on how to adjust content and the gamification principles employed in its delivery for learners to extract the maximum knowledge return for time invested. In our example, we saw a worse performance on item analysis to some of our adjusted questions used between January 2020 and November 2020. While disappointing, this underscores the need to continuously evaluate educational materials and make further adjustments as needed, with the data collected through digital gamification solutions.

Competency-based learning promotes the achievement of proficiency in critical skills, behavior, knowledge, and abilities necessary for successful job performance [19, 20]. Conversely, we propose that overall performance of a graduating class on a competency-based assessment can inform an educational institution about the effectiveness of their curricular strategies in promoting competency attainment. Learners readily and extensively participated, suggesting that we achieved a mixture of intrinsic and extrinsic motivators that successfully engaged our learners in this competition. Review of our data revealed differing global scores per competency among our existing MPH degree tracks. In multiple instances, one track outperformed another at both the >50% and >70% correct answer threshold levels of competency attainment. While some of this difference may be dependent on individual learner capabilities, the global mean scores representing the combined performance of the entire heterogeneous group of learners were similar, mitigating individual effects. A review of program level competency attainment by graduating learners should prompt discussion among faculty and leadership, triggering granular review of curricular offerings to elucidate factors contributing to different levels of competency acquisition. In our case, it provided a starting point for discussions as each track was able to assess the strengths and weaknesses of their curricula considering learner competency attainment, while gaining curricular insights from colleagues in better performing programs. This experience provides a novel framework for leveraging the data generated through a gamification-infused knowledge competition among individual learners to assess the overall efficacy of educational curricula for competency attainment. A longitudinal application of such an approach would provide a continuous quality improvement mechanism to guide ongoing curricular adjustments and educational program evaluation and optimization.

Limitations and barriers present in our experience that should be considered by all those seeking to implement gamification into their instruction include the selection of learning mechanics and game mechanics best suited to achieve specific learning objectives. There is no guarantee that the combination of learning mechanics and game mechanics used by the authors for the two described experiences would be the best suited for other situations and other groups of learners. We recommend those seeking to apply gamification principles to first analyze the target population of learners reflecting on where they are on the motivational spectrum as described by Self Determination Theory, considering which gamification strategies would most effectively guide learners toward intrinsic motivation; and second, clearly define learning objectives, then reflect on the learning mechanics to apply and which game mechanics can be leveraged to augment them using the LM-GM framework. While the gamification strategies employed in both experiences presented led to high numbers of engaged learners, we used the external motivator of a financial reward for best team and individual performances in the competency assessment game, resources which may not be available in other settings. It is important to note that the usability of our software platform exceeded the industry acceptable standard as measured in using the validated System Usability Scale (87% usability) in prior unpublished research. Finally, we assessed the foundational competencies established by our School of Public Health, and since that time, national MPH foundational competencies have been adopted. While competencies have shifted, our approach is adaptable and can be applied to the assessment of new competencies in this or other fields.

### Conclusion

We are just beginning to leverage the potential to engage learners and enhance knowledge acquisition using gamification. We urge colleagues to consider adding gamification in a variety of learning environments in a methodical way considering self-determination theory, insights on their learners and learning environments and to map their learning objectives within the LM-GM framework. Finally, we encourage all to rigorously analyze the data resulting from their experiences with gamification so that we can collectively define best practices through educational analytics, determining evidence-based insights on how to maximize educational return for time invested.
